# Draft-genome sequence of *Bacillus swezeyi* SDB04, a strain capable of synthesizing PHA at high salt concentrations

**DOI:** 10.1128/mra.00853-25

**Published:** 2026-02-04

**Authors:** Hong Wu, Yuanyuan Zhang, Lizhan Liu, Wenbo lv, Weiquan Sun, Xinyi Wen, Yilin Yang

**Affiliations:** 1 School of Life Sciences, Tianjin Normal University 12523, Tianjin, China; 2 School of Chemistry, Tianjin Normal University 12523, Tianjin, China; Nanchang University, Nanchang, Jiangxi, China

**Keywords:** *Bacillus*, draft-genome sequence, salt concentrations, PHA synthesis

## Abstract

We report the complete genome sequence of *Bacillus swezeyi* SDB04, which was isolated from a hypersaline environment and could produce Polyhydroxyalkanoates (PHA). The *de novo* assembly resulted in an estimated chromosome size of 4.29 Mb. Genomic sequence information will help to develop the potential for efficient PHA production.

## ANNOUNCEMENT

Polyhydroxyalkanoates (PHA) are linear polyesters synthesized intracellularly by many microorganisms, characterized by excellent biodegradability and biocompatibility (1, 2). Halophiles, found in high salt (NaCl) concentration areas, have been established as chassis organisms for PHA synthesis recently (3, 4). This study isolated a high-PHA-yielding halophilic strain, *Bacillus swezeyi* SDB04, from Shandong, China. Soil and water samples were collected from Chengkou Salt Field in Shandong Province (117.62° E, 37.75° N) by sterile equipment. After being prepared into suspensions, microbial cultures were carried out on a high-salt concentration screening medium. The isolation medium contained (per liter of distilled water): 20 g glucose, 60 g NaCl, 15 g MgSO₄·7H₂O, 10 g MgCl₂·6H₂O, 5 g peptone, 4 g KCl, 1 g yeast extract, 1 g CaCl₂·2H₂O, 0.5 g NaHCO₃, and 16 g agar. After being cultured aerobically at 37°C for 24 h, single colonies were picked and subjected to 0.3% (wt/vol) Sudan Black B staining to screen for potential PHA producers (5). The PHA content was subsequently quantified by gas chromatography (GC) (6), and *Bacillus swezeyi* SDB04 had the highest PHA content. The cultured cells were subjected to genomic DNA extraction and purification using the STE method. DNA integrity was verified by agarose gel electrophoresis, and purity was assessed using a NanoDrop spectrophotometer (Thermo Scientific, USA). High-quality genomic DNA was sequenced on PacBio Sequel II and Illumina NovaSeq PE150 platforms. For PacBio long reads, DNA was sheared using a Covaris g-TUBE, and a SMRTbell library was constructed with the SMRTbell Template Prep Kit (v2.0) (7). After quality control, Illumina reads were checked with FastQC v0.12.1 (8) and PacBio subreads were filtered by length/quality via SMRT Link. Assembly quality and completeness were assessed via QUAST v5.2.0 (9) and CheckM v1.2.2 (10), respectively. The optimal hybrid assembly was annotated using RAST server v2.0 (11) and Prokaryotic Genome Annotation Pipeline (12) with default parameters.

**Fig 1:**
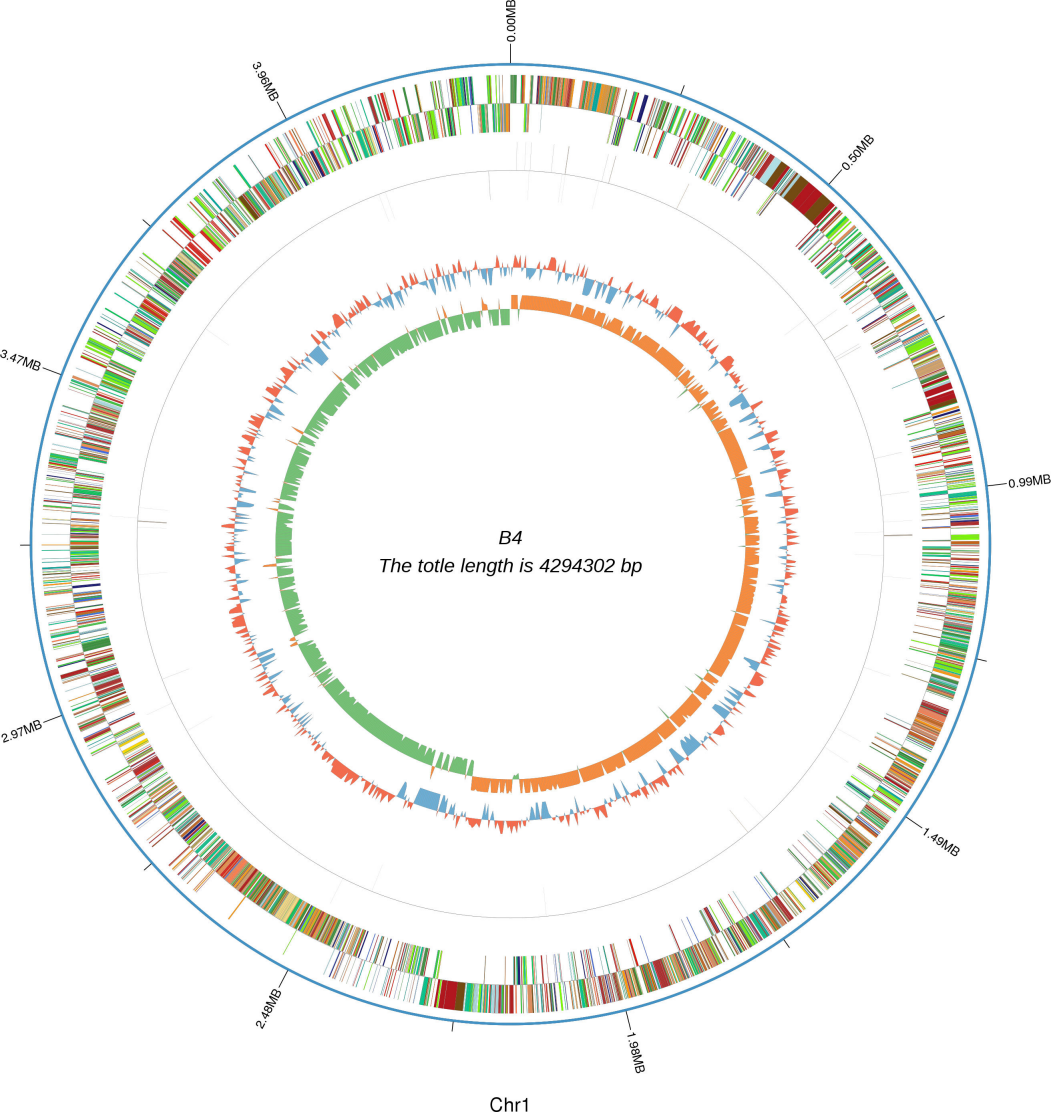
Circular representation of the *Bacillus swezeyi* SDB04 genome. This circular map illustrates the genomic characteristics of strain *Bacillus swezeyi* SDB04, which has a total genome length of 4,294,302 bp (Chromosome 1, Chr 1), and its circular tracks, ordered from the outermost to the innermost, present the following information: the outermost track displays the genomic position scale (Mb), which enables the rapid localization of specific regions on the genome and provides a coordinate reference. For the subsequent interpretation of genomic characteristics, the middle circular track shows the distribution of coding genes across different strands of the genome, where different colors or segments are used to correspond respectively to the coding genes located on the forward and reverse strands for clear distinction of their strand-specific localization; the innermost track corresponds to a GC content distribution curve, which serves to reflect the proportion of guanine (G) and cytosine (C) in each region of the genome.

Strain *Bacillus swezeyi* SDB04 had a single circular chromosome (4,294,302 bp, GC content of 44.42%) and one circular plasmid, Plas1 (65,200 bp, GC content of 46.98%) (Fig. 1). Whole-genome analysis revealed the chromosome contained 4,160 protein-coding genes (CDSs), 9 rRNA, and 52 tRNA genes (13). The total length of the coding gene sequence is 3,841,527 bp, with the average length 805 bp. The analysis of repetitive sequences by RepeatMasker (Version open-4.0.5) and Tandem Repeats Finder (14) indicated that there were 320 interspersed repeats, 78 tandem repeats included 64 minisatellite DNAs and 1 microsatellite. CRISPR Finder identified two CRISPR sequences (268 bp) (15) and genomic features comprised 13 genomic islands (16) and 4 prophages (17). By comparing and analyzing the amino acid sequences (18), a total of 2,881 functional genes were annotated to biological processes, molecular functions, and cellular components. KEGG pathway (19) enrichment mapped 4,089 genes, and Carbohydrate-Active enZyme (CAZyme) annotation (20) identified 320 enzymes, dominated by glycoside hydrolases and glycosyltransferases. After 72 h of fermentation, the PHA yield was 3.05 g/L. This study provides initial insights into the genomic characteristics of a *Bacillus swezeyi* strain capable of synthesizing PHA under high-salt conditions, providing details of metabolic pathways and related genes, laying the foundation for industrial development.

## Data Availability

From this project, *Bacillus swezeyi* SDB04 was filed under BioProject accession number PRJNA1296782 and BioSample accession numbers SAMN50199788. The raw read sequences are available under Sequence Read Archive (SRA) accession SRR34847057.

## References

[1] Paloyan A, Tadevosyan M, Ghevondyan D, Khoyetsyan L, Karapetyan M, Margaryan A, Antranikian G, Panosyan H. 2025. Biodegradation of polyhydroxyalkanoates: current state and future prospects. Front Microbiol 16. doi:10.3389/fmicb.2025.1542468 PMC11893044 40066265

[2] Chen GQ, Jiang XR. 2018. Engineering microorganisms for improving polyhydroxyalkanoate biosynthesis. Curr Opin Biotechnol 53:20–25. doi:10.1016/j.copbio.2017.10.008 29169056

[3] Tan D, Xue YS, Aibaidula G, Chen GQ. 2011. Unsterile and continuous production of polyhydroxybutyrate by Halomonas TD01. Bioresour Technol 102:8130–8136. doi:10.1016/j.biortech.2011.05.068 21680179

[4] Ren Y, Ling C, Hajnal I, Wu Q, Chen GQ. 2018. Construction of Halomonas bluephagenesis capable of high cell density growth for efficient PHA production. Appl Microbiol Biotechnol 102:4499–4510. doi:10.1007/s00253-018-8931-7 29623388

[5] Porras MA, Villar MA, Cubitto MA. 2018. Improved intracellular PHA determinations with novel spectrophotometric quantification methodologies based on Sudan black dye. J Microbiol Methods 148:1–11. doi:10.1016/j.mimet.2018.03.008 29580981

[6] Khang TU, Kim MJ, Yoo JI, Sohn YJ, Jeon SG, Park SJ, Na JG. 2021. Rapid analysis of polyhydroxyalkanoate contents and its monomer compositions by pyrolysis-gas chromatography combined with mass spectrometry (Py-GC/MS). Int J Biol Macromol 174:449–456. doi:10.1016/j.ijbiomac.2021.01.108 33485890

[7] Reiner J, Pisani L, Qiao W, Singh R, Yang Y, Shi L, Khan WA, Sebra R, Cohen N, Babu A, Edelmann L, Jabs EW, Scott SA. 2018. Cytogenomic identification and long-read single molecule real-time (SMRT) sequencing of a bardet–biedl syndrome 9 (BBS9) deletion. NPJ Genomic Med 3:3. doi:10.1038/s41525-017-0042-3 PMC5778042 29367880

[8] Brown J, Pirrung M, McCue LA, Wren J. 2017. FQC Dashboard: integrates FastQC results into a web-based, interactive, and extensible FASTQ quality control tool. Bioinformatics 33:3137–3139. doi:10.1093/bioinformatics/btx373 28605449 PMC5870778

[9] Gurevich A, Saveliev V, Vyahhi N, Tesler G. 2013. QUAST: quality assessment tool for genome assemblies. Bioinformatics 29:1072–1075. doi:10.1093/bioinformatics/btt086 23422339 PMC3624806

[10] Chklovski A, Parks DH, Woodcroft BJ, Tyson GW. 2023. CheckM2: a rapid, scalable and accurate tool for assessing microbial genome quality using machine learning. Nat Methods 20:1203–1212. doi:10.1038/s41592-023-01940-w 37500759

[11] Aziz RK, Bartels D, Best AA, DeJongh M, Disz T, Edwards RA, Formsma K, Gerdes S, Glass EM, Kubal M, et al.. 2008. The RAST Server: rapid annotations using subsystems technology. BMC Genomics 9:75. doi:10.1186/1471-2164-9-75 18261238 PMC2265698

[12] Tatusova T, DiCuccio M, Badretdin A, Chetvernin V, Nawrocki EP, Zaslavsky L, Lomsadze A, Pruitt KD, Borodovsky M, Ostell J. 2016. NCBI prokaryotic genome annotation pipeline. Nucleic Acids Res 44:6614–6624. doi:10.1093/nar/gkw569 27342282 PMC5001611

[13] Lagesen K, Hallin P, Rødland EA, Staerfeldt H-H, Rognes T, Ussery DW. 2007. RNAmmer: consistent and rapid annotation of ribosomal RNA genes. Nucleic Acids Res 35:3100–3108. doi:10.1093/nar/gkm160 17452365 PMC1888812

[14] Benson G. 1999. Tandem repeats finder: a program to analyze DNA sequences. Nucleic Acids Res 27:573–580. doi:10.1093/nar/27.2.573 9862982 PMC148217

[15] Grissa I, Vergnaud G, Pourcel C. 2007. CRISPRFinder: a web tool to identify clustered regularly interspaced short palindromic repeats. Nucleic Acids Res 35:W52–7. doi:10.1093/nar/gkm360 17537822 PMC1933234

[16] Hsiao W, Wan I, Jones SJ, Brinkman FSL. 2003. IslandPath: aiding detection of genomic islands in prokaryotes. Bioinformatics 19:418–420. doi:10.1093/bioinformatics/btg004 12584130

[17] Zhou Y, Liang YJ, Lynch KH, Dennis JJ, Wishart DS. 2011. PHAST: a fast phage search tool. Nucleic Acids Res 39:W347–52. doi:10.1093/nar/gkr485 21672955 PMC3125810

[18] Ashburner M, Ball CA, Blake JA, Botstein D, Butler H, Cherry JM, Davis AP, Dolinski K, Dwight SS, Eppig JT, Harris MA, Hill DP, Issel-Tarver L, Kasarskis A, Lewis S, Matese JC, Richardson JE, Ringwald M, Rubin GM, Sherlock G. 2000. Gene Ontology: tool for the unification of biology. Nat Genet 25:25–29. doi:10.1038/75556 10802651 PMC3037419

[19] Kanehisa M, Goto S, Kawashima S, Okuno Y, Hattori M. 2004. The KEGG resource for deciphering the genome. Nucleic Acids Res 32:D277–80. doi:10.1093/nar/gkh063 14681412 PMC308797

[20] Drula E, Garron ML, Dogan S, Lombard V, Henrissat B, Terrapon N. 2022. The carbohydrate-active enzyme database: functions and literature. Nucleic Acids Res 50:D571–D577. doi:10.1093/nar/gkab1045 34850161 PMC8728194

